# Inherited Retinal Degeneration: Genetics, Disease Characterization, and Outcome Measures

**DOI:** 10.1155/2017/2109014

**Published:** 2017-09-25

**Authors:** Naheed W. Khan, Benedetto Falsini, Mineo Kondo, Anthony G. Robson

**Affiliations:** ^1^Department of Ophthalmology & Visual Science, University of Michigan, Ann Arbor, MI 48105, USA; ^2^Department of Ophthalmology, Fondazione Policlinico Universitario A. Gemelli, Università Cattolica del S. Cuore, Rome, Italy; ^3^Department of Ophthalmology, Mie University Graduate School of Medicine, Tsu, Japan; ^4^Moorfields Eye Hospital, 162 City Road, London EC1V 2PD, UK; ^5^Institute of Ophthalmology, University College London, 11-43 Bath Street, London EC1V 9EL, UK

Inherited retinal degenerations (IRDs) lead to incurable vision loss and affect 1 in 2000 to 3000 individuals. These disorders may cause blindness associated with dysfunction or death of photoreceptor cells, but prognosis is difficult to determine due to variable disease expression. There have been significant advances in understanding the pathogenesis and genetics of IRDs with more than 250 genes being implicated to date. This has led to the development of treatments aimed at restoring function or delaying progression.

The design of therapeutic interventions depends on the phenotype and the molecular characteristics of the IRD. With advances in genetic testing methodology, there is an increased detection of variants in multiple genes in the same family or even in the same individual, which makes the determination of the primary genetic cause of disease more difficult to assess. Using next generation sequencing, Meng et al. show in a multigeneration Chinese family that *PRPF3*-associated autosomal dominant retinitis pigmentosa coexists with *CYP4V2*-associated Bietti's crystalline corneoretinal dystrophy. This further complicates the characterization of phenotypes of an autosomal dominant condition with an autosomal recessive condition which results in a more severe disease phenotype.

Due to genetic heterogeneity of these retinal conditions, patients may have very similar clinical phenotypes but different genetic diagnoses, requiring for example gene-specific therapy or gene-editing treatments to address the underlying cause of disease. Development of effective treatment strategies requires robust clinical characterization to establish genotype-phenotype correlations and to determine the therapeutic window and optimal stage for intervention. Katagiri and colleagues demonstrate in 20 patients with angioid streaks that *ABCC6* variants play a significant role in affected Japanese individuals. Retinal degenerations may express variability in phenotype even within the same family. Iarossi et al. report genetic heterogeneity and associated variable phenotypes in familial exudative vitreoretinopathy (FEVR) which is a complex disorder characterized by incomplete development of the retinal vasculature. The phenotypic variability in such disorders adds a further degree of complexity when establishing study protocols.

Another major consideration is the development of tests to monitor the natural history of the disease to best measure outcomes and to evaluate therapeutic efficacy in human clinical trials. Newer imaging technologies have made it possible to study microstructural details of the retina. Quantitative assessment of the structural and functional integrity of the retina and the correlation between the functional and structural measures will improve our understanding of the disease and help in the design of appropriate outcome measures and therefore in determining which patients might be the best candidates for treatments. There is a battery of methods to test visual function and retinal function, which poses a challenge when deciding which test or which combination of tests to choose for a particular phenotype. Abed and colleagues evaluate the relationship between cone photoreceptor function assessed by visual acuity and the focal electroretinogram (FERG) and the integrity and structure of photoreceptors using optical coherence tomography in Stargardt disease. Their study demonstrates that FERG amplitude can be reliably used to monitor macular cone function and that visual acuity is a useful indicator of foveal function.

Neuroprotection is a largely nonspecific strategy to provide a protective environment for slowing the process of photoreceptor degeneration, thus extending the therapeutic window to later stages of disease. Exercise has been shown to have neuroprotective effects on photoreceptors in mouse models of retinal degeneration. Levinson et al. did a retrospective case-control study to report baseline physical activity levels in individuals with retinitis pigmentosa. They used three quality of life questionnaires to determine the relationship between physical activity and visual function and showed that increased physical activity is associated with greater self-reported visual function and quality of life.

*Naheed W. Khan* *Benedetto Falsini* *Mineo Kondo* *Anthony G. Robson*

